# Effect of three different insect larvae on growth performance and antioxidant activity of thigh, breast, and liver tissues of chickens reared under mild heat stress

**DOI:** 10.1007/s11250-024-03923-1

**Published:** 2024-02-15

**Authors:** Vasilopoulos Stelios, Giannenas Ilias, Panitsidis Ioannis, Athanassiou Christos, Papadopoulos Elias, Fortomaris Paschalis

**Affiliations:** 1 Laboratory of Nutrition, Faculty of Veterinary Medicine, Aristotle University of Thessaloniki, 54124 Thessaloniki, PC Greece; 2 Laboratory of Entomology and Agricultural Zoology, Department of Agriculture, Crop Production and Rural Environment, University of Thessaly, Phytokou Str., 38446 Volos, N. Ionia Greece; 3 Laboratory of Parasitology and Parasitic Diseases, Faculty of Veterinary Medicine, Aristotle University, 54124 Thessaloniki, Greece; 4 Laboratory of Animal Husbandry, Faculty of Veterinary Medicine, Aristotle University of Thessaloniki, 54124 Thessaloniki, Greece

**Keywords:** Broiler, Insect meal, Heat stress, Mealworm, Black soldier fly, Superworm

## Abstract

This study investigated the potential of insect-based diets to mitigate heat stress impact on broiler chickens, focusing on growth performance and antioxidant stability. Four dietary groups were examined, including a control and three treated groups with *Tenebrio molitor* (TM), *Hermetia illucens* (HI), and *Zophobas morio* (ZM) larvae, respectively, at a 5% replacement ratio. Temperature and relative humidity of the poultry house were monitored. Under heat stress conditions, the HI-fed group consistently exhibited the highest body weight, demonstrating their remarkable growth-promoting potential. TM-fed broilers also displayed commendable growth compared to the control. Insect larvae inclusion in the diet improved feed intake during early growth stages, indicating their positive influence on nutrient utilization. Regarding antioxidant stability, malondialdehyde (MDA) levels in the liver, an oxidative stress and lipid peroxidation marker, were significantly lower in the TM-fed group, suggesting reduced oxidative stress. While the specific insect-based diet did not significantly affect MDA levels in thigh and breast tissues, variations in the total phenolic content (TPC) were observed across tissues, with HI larvae significantly increasing it in the breast. However, the total antioxidant capacity (TAC) and 2,2-diphenyl-1-picrylhydrazyl (DPPH) levels did not differ significantly among dietary groups in the examined tissues. Results suggest that insect-based diets enhance broiler growth and potentially reduce oxidative stress, particularly in the liver. Dietary presence of bioactive compounds may contribute to these benefits. Further research is required to fully elucidate the mechanisms underlying these findings. Insect-based diets seem to offer promise as feed additives in addressing the multifaceted challenges of oxidative stress and enhancing broiler health and resilience under heat stress conditions.

## Introduction

In recent years, an increase in global demand for poultry products has emerged, particularly broiler chicken, and has witnessed a remarkable surge due to its high nutritional value and widespread consumer appeal (FAO 2022). It is understandable that the poultry industry faces the challenge of ensuring a sustainable and cost-effective supply of feed to meet this escalating demand. To address this challenge, researchers and stakeholders have been exploring alternative protein sources that are both environmentally friendly and economically viable (Giannenas et al. 2017). Among these alternatives, the use of insects in broiler feed has emerged as a promising and innovative solution (Allegretti et al. 2018; Shah et al. 2022). The integration of insect-based diets not only addresses the nutritional needs of broiler chickens but also offers a potential environmental footprint reduction of the poultry industry. This approach aligns with the growing emphasis on sustainable and eco-friendly practices within the poultry sector (Heines et al. 2022).

Insects, such as *Hermetia illucens* (HI, black soldier fly larvae), *Tenebrio molitor* (TM, yellow mealworm), and *Zophobas morio* (ZM, superworm) and their larvae, provide a sustainable and environmentally friendly supply of protein and fat, making them an attractive option for poultry nutrition. They have a high protein content and contain essential amino acids, making them a valuable replacement for traditional protein sources like soybean and fishmeal (Lu et al. 2022; Nowak et al. 2016). There is, however, an overestimation of the true protein content of insects which, notwithstanding the comparable levels to traditionally used commodities like soybean meal, it should be preferably calculated with conversion factors closer to 5.6 or even lower (Boulos et al. 2020).

Additionally, in comparison to conventional animal farming, insect farming has a substantially lower environmental effect, requiring less land, water, and feed inputs. The utilization of insects in broiler feed not only reduces the dependence on scarce resources but also contributes to lower greenhouse gas emissions and mitigates the adverse environmental impacts associated with conventional feed sources. Moreover, insects can be reared on organic side streams, transforming them into valuable nutrients, promoting the concept of a circular economy (Halloran et al. 2017). It is important to clarify, however, that insects, currently, can only be fed with vegetable origin materials with some specific exceptions analytically described in Regulation (EU) 2022/1104. Catering waste, manure, and slaughterhouse or rendered by-products are clearly prohibited. A recent comprehensive review by Yang et al. (2023) summarizes edible insect nutritional characteristics and possible applications in the food industry, interestingly focusing on their protein immune responses, which also involve allergenicity. As research and commercial implementation continue to progress, the incorporation of insects into broiler feed holds great promise as a sustainable and nutritionally beneficial alternative for the poultry industry.

Except for their high-quality amino acid and fatty acid profile, insects used as feed for broiler chickens offer an additional advantage through their abundance of natural antioxidants, including vitamins E and C, carotenoids, and phenolic compounds (Torres-Castillo and Olazarán-Santibáñez 2023; Zulkifli et al. 2022). When broiler chickens consume insect-based feed, these antioxidants are effectively transferred to their muscle tissues, enriching the meat with these beneficial compounds (Gullan and Cranston 2014; Köhler et al. 2019; Paoletti 2005; Rumpold and Schlüter 2013). These natural antioxidants play a critical role in safeguarding cells and tissues from oxidative damage caused by free radicals. Consequently, the chicken meat becomes more resistant to lipid oxidation, preserving its freshness and extending its shelf life. Moreover, the presence of these antioxidants contributes to improved meat color stability, tenderness, and overall nutritional value.

Besides its advantageous nutritional composition, insects as food or feed additives may support broilers to overcome the challenge of heat stress. Stress, a complex response to adverse stimuli, often eludes precise description due to its multifaceted nature. Friend (1980) aptly defines stress in animals as an observable reaction to circumstances that necessitate unusual or significant changes in their behavior or physiology to contend with unfavorable aspects of their environment. Consequently, stress manifests as the organism’s adaptive response to stimuli that disrupt its normal physiological equilibrium or homeostasis.

As global temperatures continue to rise due to global warming, broilers are becoming increasingly susceptible to heat stress, a condition that can have profound and adverse effects on their health and performance. Heat stress occurs when broilers are exposed to high temperatures and humidity levels, exceeding their thermal comfort zone (Tawfeek et al. 2014). This environmental stressor can trigger a cascade of physiological responses, including inflammation. When broilers experience heat stress, their bodies release pro-inflammatory molecules as part of their natural stress response. This inflammation is a defense mechanism aimed at regulating body temperature and restoring homeostasis (Cantet et al. 2021). In general, the comfort zone for broilers fluctuates between 16 and 26 °C (Diarra and Tabuaciri 2014). Mascarenhas et al. (2020) have listed the ideal temperature values for different broiler life stages, namely 28–34 °C for the starter, 24–28 °C for the grower, and 18–24 °C for the finisher periods. In this trial, the thermal comfort zone was exceeded during the grower and finisher periods.

Thermal comfort zones are measured by thermal comfort indices, and according to the animal species, a major element which drives the heat exchange between the environment and an animal is the dry-bulb temperature and represents the sensible heat content of the air. Apart from that, other environmental parameters impact the total heat exchange as well (relative humidity, RH, thermal radiation, airflow) (Hahn et al. 2013). The temperature-humidity index (THI) has been extensively used and quantifies the degree of heat stress on broilers by using both the temperature and RH.

However, prolonged or severe heat stress can lead to excessive and chronic inflammation, which is detrimental to broiler health. The inflammatory response can compromise the integrity of the intestinal barrier, leading to leaky gut syndrome (Elnesr and Abdel-Azim 2023; Karl et al. 2018). This condition allows harmful bacteria and toxins to enter the bloodstream, potentially causing systemic inflammation and immune system activation. Inflammatory responses can also affect various organs and tissues in broiler chickens. For instance, the liver may experience inflammation, impairing its metabolic functions, including nutrient utilization and detoxification (Ma et al. 2022). This can further exacerbate heat stress’s harmful influence on broiler performance. Furthermore, oxidative stress often accompanies heat stress–induced inflammation. The excessive generation of reactive oxygen species (ROS) can overwhelm the antioxidant defense mechanisms of broiler chickens (Xie et al. 2015). This imbalance can result in oxidative damage to cells, proteins, and DNA, contributing to reduced feed intake (FI), decreased body weight gain (BWG), and increased mortality rates (MR) in broilers.

Heat stress disrupts the body’s ability to regulate temperature, leading to panting in broiler chickens. This panting response can further contribute to respiratory challenges and dehydration. A study by Mutibvu et al. (2017) observed that for each incremental unit increase in the temperature-humidity index, broiler chickens exhibited an additional 0.56 breaths per minute, highlighting the direct impact of environmental heat stress on avian physiology. By incorporating insects into the diet of broiler chickens, with their natural antioxidants, balanced amino acid, and fatty acid profiles, the resilience of broilers may be enhanced against heat stress. These insect-based diets can support overall health and help mitigate oxidative damage caused by high temperatures and inflammation.

Expanding upon the encouraging potential of insect-based diets and their ability to alleviate the negative impacts of heat stress on broiler chickens, a critical research gap emerges in comprehending how these diets specifically influence the performance parameters and oxidative status under conditions of mild heat stress. This research seeks to address this gap by investigating the precise mechanisms through which insect-based diets impact the performance outcomes of broiler chickens under mild heat stress, particularly focusing performance parameters such as BWG, feed conversion ratios (FCR), and MR. The study aims to explore the interactions between natural antioxidants inherent in these insects and the oxidative stress induced by heat, providing insights into their role in maintaining optimal oxidative status. Additionally, it will examine how these diets influence performance parameters, shedding light on their potential to enhance weight gain, feed efficiency, and reduce MR when broilers are exposed to mild heat stress conditions.

## Materials and methods

### Ethics approval

The study properly followed Greek law on the use of experimental animals. These standards were strictly followed in all elements of animal husbandry, euthanasia, experimental methods, and biosecurity precautions. The study protocol has been approved by the Aristotle University Study Committee in Thessaloniki, Greece, with registration number (928/17–1-2020). The local Public Veterinary Authorities have approved these procedures [Reg. 489,181 (3254)/07.02.2018].

### Experimental design diets and housing

In this study, 240-day-old commercial male Ross 308 broiler chicks were individually weighed and divided into four nutritional treatment groups, each with six replicates of ten birds. The chicks were reared under optimal conditions until they reached 10 days of age. Subsequently, they were subjected to a heat stress challenge. The temperature was gradually reduced from 36 °C on day 1 to 22 °C on day 14 and remained stable until day 24, when the heat stress regimen was initiated. This regimen involved a daily 6-h program with the temperature maintained at 28 °C. The lighting schedule initially provided 24 h of continuous light until the second day of the experiment, after which it was reduced to 23 h per day, following the guidelines set by the breeding company (Aviagen®). A veterinarian checked on the animals’ health once a day. The experiment took place at the Hellenic Agricultural Organization—Dimitra in Giannitsa, Greece, and each treatment was preserved in a specifically built chamber. The housing consisted of a dedicated area containing 24 pens, each measuring 120 × 120 cm. Each pen was equipped with a hanging feeder and a nipple drinker with a bedding of straw. On the first day at the hatchery, birds received vaccinations against infectious bursal disease (IBD), infectious bronchitis (IB), and Newcastle disease (ND).

Insects used were reared in the University of Thessaly facilities, Greece, at the Laboratory of Entomology and Agricultural Zoology, at 30 °C and 65 RH. The substrate used was 90% wheat and 10% yeast, and humidity was offered via a 1% agar gel. Larvae were sieved and separated after 60–65 days at their optimum size. They were then dried in an electric dryer (Ventolab® Agro HW 6/14tray dryer, Vencon Varsos S.A.) and stored before being used as part replacement of the basal diet. Control and treated diet composition, including moisture, crude protein, ether extract, crude fiber, starch, ash, calcium, phosphorus, and amino acids, was analyzed using a PerkinElmer DA 7250 At-Line NIR Perten analyzer, which is part of the third generation of NIR diode instruments intended expressly for use in the food and agriculture sectors. Table 1 summarizes the findings. The diets were formulated according to Ross 308 Standard Management Recommendations (Aviagen 2014). The control contained the basal diet, and according to that, additional diets were created by adding either TM at 50 g/kg feed in treatment one, HI at 50 g/kg feed in treatment two, and ZM at 50 g/kg feed in treatment three.

**Table 1:** Basal and experimental diets composition in the three growing periods

Period	*Diet composition*											
	Control			*Tenebrio molitor* (TM)			*Hermetia illucens* (HI)			*Zophobas morio* (ZM)		
	Starter	Grower	Finisher	Starter	Grower	Finisher	Starter	Grower	Finisher	Starter	Grower	Finisher
Measured nutrient content, % as is*												
Moisture	10.01	10.10	9.71	9.90	9.82	9.68	9.69	9.37	9.90	9.28	9.16	8.95
Crude protein	21.70	20.29	19.79	22.62	21.18	20.67	23.21	21.84	21.27	22.82	21.38	20.90
Ether extract	3.35	4.11	5.16	3.86	4.59	5.70	3.94	4.75	5.77	4.01	4.80	5.70
Crude fiber	3.15	3.20	3.28	3.50	3.80	3.30	3.33	3.24	3.31	3.54	3.26	3.37
Starch	39.99	40.55	39.70	39.54	39.44	39.14	38.88	39.36	38.63	36.67	36.27	36.87
Ash	8.32	8.48	7.89	8.16	8.75	8.71	8.64	9.26	9.14	8.66	8.56	8.82
Calcium	0.90	0.81	0.65	1.00	1.22	1.01	1.06	1.40	0.80	1.16	1.50	0.98
Phosphorus	0.61	0.64	0.59	0.77	0.56	0.80	0.66	0.81	0.93	0.75	0.76	0.56
*Amino acids, % dry basis*												
Threonine	1.27	1.21	1.33	1.44	1.39	1.45	1.64	1.62	1.69	1.27	1.32	1.31
Glutamic acid	3.91	4.26	3.94	9.75	9.84	9.31	9.34	9.73	9.47	7.95	7.58	7.98
Alanine	1.51	1.51	1.48	2.39	2.43	2.37	2.24	2.27	2.28	1.58	1.54	1.52
Cysteine	0.49	0.55	0.53	0.17	0.16	0.13	0.22	0.16	0.28	0.25	0.31	0.29
Valine	1.43	1.47	1.46	2.80	2.86	2.77	2.43	2.48	2.48	1.73	1.67	1.73
Methionine	0.55	0.50	0.52	0.36	0.40	0.36	0.36	0.39	0.40	0.18	0.24	0.20
Isoleucine	1.26	1.32	1.30	2.52	2.58	2.47	2.80	2.81	2.81	2.14	2.10	2.10
Leucine	2.16	2.06	2.34	3.56	3.75	3.44	3.58	3.75	3.71	2.68	2.62	2.56
Tyrosine	0.79	0.81	0.84	1.48	1.45	1.53	1.67	1.71	1.64	1.77	1.76	1.74
Phenylalanine	1.23	1.20	1.22	2.63	2.68	2.62	2.81	2.78	2.81	2.42	2.47	2.40
Lysine	2.06	2.05	1.91	2.32	2.40	2.50	1.87	1.87	1.79	1.59	1.74	1.72
Histidine	0.89	0.83	0.87	1.44	1.48	1.41	1.20	1.16	1.24	0.80	0.83	0.75
Arginine	1.82	1.76	1.85	4.03	4.10	4.05	3.39	3.30	3.33	2.41	2.34	2.34
Tryptophan	0.57	0.60	0.59	0.37	0.38	0.40	0.89	0.89	0.86	0.64	0.68	0.59

^*^Ingredients added in mash form

### Environmental data collection

Environmental data during the trial were collected. A temperature-humidity record system (HOBO UX100-003 Temperature/RH data logger, Onset Computer Corporation, Bourne, USA) was used to monitor the temperature and RH. The data logger was placed in the immediate breeding environment and hung on the wall of the poultry house. Logged data was collected on a weekly basis and used to calculate THI, as a measure of the thermal comfort of the broilers. THI was calculated based on the equation described by Omomowo (2021).

### Performance parameters

Throughout the trial, for each diet treatment throughout the whole study period health condition, MR, body weight (BW), BWG, FI, and FCR were tracked and calculated on a three-period basis, the starter period (days 1–10), the grower period (days 11–24), and the finisher period (days 25–35). All measurements were taken using a high accuracy electronic scale (CAS model SW-1, Korea).

### Antioxidant status

Two birds selected at random from each pen were euthanized and defeathered. Breast and thigh muscles as well as liver samples were removed from the carcass, weighted, and stored at − 80 °C until analyzed.

Malondialdehyde (MDA) was used as a marker to measure lipid oxidation, according to Ahn et al. (1999). 0.5 g tissue samples were homogenized in hexane with 4 ml 5% trichloroacetic acid (TCA) and 2.5 ml 0.8% butylated hydroxytoluene (BHT). After centrifugation at 3000 g for 5 min, 1.5 ml of the underlying layer was recovered. The mixture was then incubated in a water bath at 70 °C for 30 min with 2.5 ml of 0.8% thiobarbituric acid (TBA). The absorbance of the final sample was measured using a spectrophotometer at 532 nm. MDA concentrations in the samples were measured by constructing a reference curve with known amounts of MDA and expressing the results in nmol/mg.

The assessment of the total phenolic content (TPC) in chicken meat tissue was conducted following a modified procedure based on the method established by Jang et al. (2008). In 6 ml of distilled water, 2 g of tissue was homogenized. After that, 3.6 ml of dichloromethane was added to the homogenate and vortexed. The supernatant was collected and measured after centrifuging the resultant sample. To create the diluted sample, 1 ml of supernatant was mixed with 4 ml of distilled water. The diluted sample was then combined with 500 µl of Folin-Ciocalteu reagent and 1 ml of 7% Na2CO3 solution. A vortex was used to thoroughly blend the mixture, which was then incubated at room temperature for 60 min. The TPC was assessed after incubation using a spectrophotometer calibrated to detect absorbance at 700 nm.

The total antioxidant capacity (TAC) was assessed following the method described by Prieto et al. (1999) with some minor modifications, using a phosphomolybdate reagent. After vortexing 100 µl of tissue extracts with 1 ml of the phosphomolybdate reagent, the mixture was left to incubate for 90 min at 95 °C in a water bath. Using a spectrophotometer, the absorbance was determined at 695 nm after cooling. Ascorbic acid was used to create a standard sample, and phosphomolybdate reagent was used to create a blank sample in the absence of the tissue sample. The TAC was calculated by the following formula: antioxidant activity = (Abs sample − Abs blank)/(Abs standard − Abs blank) × 100.

Protein oxidation status was measured using the method described by Patsoukis et al. (2004) to determine protein carbonyls in chicken tissue samples. To initiate the process, 50 µl of a 20% solution of TCA was introduced to 50 µl of the homogenized sample, which had been previously diluted in a 1:2 ratio (sample volume to diluent volume). This mixture was then placed in an ice bath for incubation and subsequently subjected to centrifugation. The resulting liquid portion (supernatant) was discarded, leaving behind a solid residue, to which 2,4-dinitrophenylhydrazine (DNPH) was added. After allowing this mixture to incubate at room temperature in the absence of light, it underwent another round of centrifugation. Once again, the liquid portion (supernatant) was removed, and the remaining pellet was exposed to 1 ml of 10% TCA, followed by thorough mixing and centrifugation. The supernatant was then disposed of, and 1 ml of a mixture containing ethanol and ethyl acetate in a 1:1 ratio (v/v) was added to the pellet. After vortexing and subsequent centrifugation, the supernatant was carefully decanted, and the remaining pellet was treated with 1 ml of a solution containing 5 mol/l urea with a pH of 2.3. The mixture was once again vortexed and incubated at 37 °C for 15 min, followed by centrifugation at 15,000 g for 3 min at 4 °C. In this particular assay, the presence of protein carbonyls was detected by their reaction with DNPH, resulting in the formation of 2,4-dinitrophenylhydrazone (DNP-hydrazone). The absorbance of DNP-hydrazone was then measured at 375 nm. The concentration of protein carbonyls was determined using the molar extinction coefficient of DNPH and expressed as nmol/mg.

Finally, to measure the radical scavenging activity in chicken tissue, a 25 µl aliquot of the sample was blended with 975 µL of 2,2-diphenyl-1-picrylhydrazyl (DPPH) solution (100 µM in MeOH) as described by Vasilopoulos et al. (2022). After the mixture had been left in the dark for a 30-min incubation, a UV–Vis spectrophotometer was used to detect the absorbance at 515 nm. DPPH solution was used to create a control that had no sample. The percentage of radical scavenging activity (% RSA) for each chicken tissue sample was calculated using the formula: (% RSA) = [(Ablank − Asample)/Ablank] × 100.

### Statistical analysis

The replication (pen) was used as the experimental unit in the RCBD (random complete block design) investigation. The minimum needed total sample size was computed before the start of the experiment using the “Power analysis for one-way ANOVA” approach (Charan and Kantharia 2013) with G*Power 3.1.9.2 software (Faul et al., Universitat Kiel, Germany) and power 0.80. Analysis of variance (ANOVA) was performed on experimental data using the statistical package of SPSS software v.27.0.1 (SPSS Inc./IBM Corp., Armonk, New York, NY, USA), using ANOVA (Tukey’s post hoc test). The threshold for statistical significance (*p*) was established at 0.05%.

## Results

### Housing environment

**Fig. 1:**
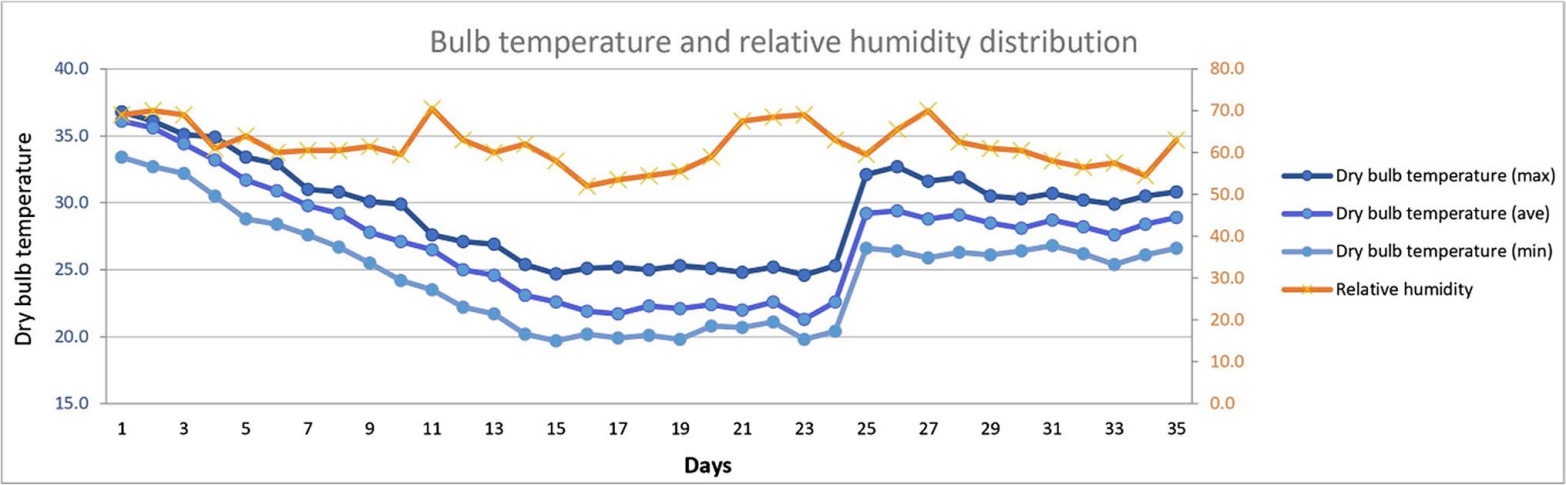
Dry-bulb temperature and relative humidity distribution for the duration of the experiment

Climatic data in the facility was monitored throughout the experimentation period. Ambient temperature and RH were measured on a daily basis, as shown in Fig. 1.

The min, average, and max daily temperature outside the poultry house were 14.2 °C, 18.3 °C, and 28.4 °C, respectively, during the trial. The calculated THI is given in Table 2.

**Table 2:** Calculated THI (°C) during the experiment

	DAYS																																		
	*1*	*2*	*3*	*4*	*5*	*6*	*7*	*8*	*9*	*10*	*11*	*12*	*13*	*14*	*15*	*16*	*17*	*18*	*19*	*20*	*21*	*22*	*23*	*24*	*25*	*26*	*27*	*28*	*29*	*30*	*31*	*32*	*33*	*34*	*35*
THI (°C)	34.0	33.6	32.5	30.9	29.8	28.9	27.9	27.4	26.2	25.5	25.4	23.8	23.3	22.1	21.5	20.8	20.6	21.2	21.0	21.4	21.2	21.8	20.6	21.7	27.3	27.8	27.5	27.4	26.8	26.4	26.8	26.3	25.9	26.4	27.2

### Growth performance

The data for broiler chicken growth performance is summarized in Table 3. This table provides insights into how different dietary treatments, specifically the inclusion of various insect species (TM, HI, ZM), influenced the BW of the broiler chickens at different time points during the experiment.

**Table 3:** The effect of insect supplementation on BW, BWG, FI, and FCR in broilers under mild heat stress

Performance indicator	Groups				SEM	*p*-value
	Control (C)	*Tenebrio molitor* (TM)	*Hermetia illucens* (HI)	*Zophobas morio* (ZM)		
BW^1^						
Day 1	42.4	42.6	42.3	42.1	0.93	0.293
Day 10	126.1^c^	150.7^ab^	163.1^a^	137.2^bc^	4.58	0.002
Day 24	531.7^b^	674.5^a^	752.7^a^	655.9^ab^	27.07	0.005
Day 35	1348.3^b^	1570.7^ab^	1710.8^a^	1582.5^ab^	48.12	0.026
BWG^1^						
Day 1–10	83.8^c^	108.2^ab^	121.5^a^	95.3^bc^	4.68	0.002
Day 10–24	406.1^b^	523.8^ab^	588.8^a^	518.6^ab^	22.73	0.007
Day 24–35	816.7	894.4	968.0	926.6	24.11	0.136
Day 1–35	1305.9^b^	1528.5^ab^	1668.5^a^	1540.4^ab^	48.12	0.026
FI^1^						
Day 1–10	100.03	125.63	124.43	110.87	3.28	0.109
Day 10–24	571.57^b^	709.20^ab^	741.27^a^	705.67^ab^	24.09	0.026
Day 24–35	1416.73	1471.23	1538.97	1537.47	31.59	0.517
FCR						
Day 1–10	1.19	1.16	1.08	1.16	0.04	0.481
Day 10–24	1.41	1.36	1.26	1.37	0.04	0.619
Day 24–35	1.74	1.66	1.59	1.66	0.05	0.790
Day 1–35	1.60	1.52	1.44	1.53	0.05	0.753

^a,^^b^Superscripts in the same row indicate means vary (*p* < 0.05)

^1^Measured in grams

*BW* body weight, *BWG* body weight gain, *FI* feed intake, *FCR* feed conversion ratio, *SEM* standard error of means

On the first day of the experiment, there were no significant differences in BW among the groups. However, as the experiment progressed, distinct patterns in BW began to emerge. By day 10, significant differences became evident. The group fed with HI larvae displayed the highest BW, with a mean of 163.07 g, which was significantly higher than the other groups. This group was followed closely by the TM-fed group. In contrast, the control had the lowest BW at this stage. The ZM-fed group had an intermediate BW. These trends continued to day 24 and day 35. At both time points, the HI-fed group consistently had the highest BW, followed by the TM-fed group. These two groups significantly outperformed the control and ZM-fed groups in terms of BW. The differences were statistically significant, as indicated by the associated *p*-values (*p* = 0.005 for day 24 and *p* = 0.026 for day 35). Results suggest that dietary supplementation with HI and TM larvae positively influenced the BW of broiler chickens compared to the control and ZM-fed groups. HI-fed chickens consistently exhibited the highest BW throughout the experiment, indicating the potential benefits of this dietary treatment for promoting growth in broiler chickens.

In terms of BWG, Table 3 provides valuable insights into the performance of these groups. During the initial period from day 1 to day 10, it is evident that the HI-fed group exhibited the highest BWG, significantly outperforming the ZM-fed and control. The TM-fed group also displayed a respectable BWG (108.19), and it was significantly higher than the control which had the lowest BWG (83.82). The ZM-fed group falls in between the control and TM-fed groups, with a moderate BWG. In the second interval from day 10 to day 24, a similar pattern emerges. Once again, the HI-fed group stands out with the highest BWG, which was significantly higher than the control. The TM-fed and ZM-fed groups show intermediate growth. The control once again was the one with the lower BWG. In the third interval from day 24 to day 35, all groups demonstrate substantial increases in BWG. However, there are no statistically significant differences among the groups during this period, as indicated by the *p*-values (0.136).

Throughout the entire treatment period, a statistically significant variation in BWG among the groups was observed (*p* = 0.026). These findings underscore the influence of the specific insect-based diets, represented by TM, HI, and ZM on the growth and weight gain of the subjects when compared to the control. Remarkably, the HI-fed group emerges as the frontrunner, demonstrating the most substantial BWG, which is significantly greater than that of the control. On the other hand, the TM-fed and ZM-fed groups exhibit intermediate weight gains, and although they do not display statistically significant differences from the control, they do show a noticeable trend towards improved weight gain. Results reveal that, over the course of this study, the HI group consistently exhibited the highest BWG across the two first periods. During these stages, TM and ZM groups also displayed decent growth, outperforming the control. However, by the last period, there were no significant differences in BWG among the groups, suggesting that they all reached a similar growth stage.

FI across the insect-fed groups displayed in Table 3 shows that during the initial period from day 1 to day 10 as well as the final period from day 24 to day 35, there were no significant differences in FI among the groups. All groups consumed feed within a similar range, with no statistically significant variations. However, from day 10 to day 24, notable differences in FI emerged among the groups. The HI-fed group exhibited the highest FI during this interval, (*p* = 0.026) when compared to the control. TM- and ZM-fed groups also showed increased feed consumption during this period, although their intake levels were not significantly different from the control.

Lastly, Table 3 provides insights into the feed conversion ratio (FCR). During all phases of the experiment, the HI group showed the most efficient feed conversion, with the control exhibiting the least efficient conversion. However, as the experiment progressed into the second and last periods, FCR values among the groups became more consistent, with no significant differences observed.

As heat stress casts a major concern in poultry businesses, affecting performance and incurring major economic losses, MR was monitored throughout the trial. There was no mortality evident in the HI and TM groups. Strain did not affect mortality as only two cases were monitored on days 22 and 32, in the control and ZM groups, respectively. These results are not indicators of a reduced ability of the chicken to withstand mild heat stress.

### Antioxidant stability

Table 4 presents the results of the experiment measuring the levels of MDA, TPC, TAC (%), and DPPH (%) in different tissue samples (thigh, breast, and liver) of the different groups (control, TM, HI, and ZM). MDA is used as an indicator of oxidative stress and lipid peroxidation, while TPC is a measure of the concentration of phenolic compounds, which are a class of secondary metabolites found in plants and some insects; phenolic compounds are known for their antioxidant properties and can have various health benefits. TAC, on the other hand, is a measure of the ability of a substance to neutralize oxidative stress and free radicals, indicating the antioxidant capacity of these tissues, and was determined with the phosphomolybdate method. The antioxidant activity was assessed by the DPPH method (%), which gauges the capacity of a substance, typically an antioxidant or radical scavenger, to counteract or diminish DPPH radicals.

**Table 4:** The effect of insect supplementation on MDA, total phenolic content, total antioxidant capacity, and on DPPH in broilers under mild heat stress

Studied parameter	Groups				SEM	*p*-value
	Control (C)	*Tenebrio molitor* (TM)	*Hermetia illucens* (HI)	*Zophobas morio* (ZM)		
MDA in ng/g sample						
Thigh	5.64	2.59	3.82	7.69	0.87	0.183
Breast	6.18	2.47	3.64	9.01	1.19	0.219
Liver	12.14^a^	6.98^b^	9.34^ab^	11.23^ab^	0.73	0.048
Total phenolic content in µg GAE/g DW						
Thigh	233.7^a^	225.2^a^	239.1^a^	103.1^b^	12.74	< 0.001
Breast	265.6^b^	271.1^b^	477.3^a^	265.0^b^	22.70	< 0.001
Liver	315.4^b^	382.8^ab^	457.2^a^	356.0^ab^	19.56	0.050
Antioxidant capacity (%)						
Thigh	33.1	28.5	30.1	30.0	1.59	0.740
Breast	34.1	32.8	34.3	31.2	1.57	0.907
Liver	46.4	54.6	53.0	46.3	1.70	0.178
DPPH in % RSA						
Thigh	17.42	16.31	15.11	16.63	1.2	0.946
Breast	17.04	16.7	14.62	13.72	1.23	0.791
Liver	55.07	57.23	58.59	56.45	2.18	0.942

^a,^^b^Superscripts in the same row indicate means vary (*p* < 0.05)

*MDA* malondialdehyde, *ng/g* nanograms/grams, *µg GAE/g DW* micrograms of Gallic acid equivalent per gram of dry weight, *DPPH* 2,2-diphenyl-1-picrylhydrazyl, *RSA* radical scavenging activity, *SEM* standard error of means

MDA levels in the thigh tissue of the different groups show some variations, but these differences are not statistically significant (*p* > 0.05). This suggests that there may not be a substantial difference in lipid peroxidation or oxidative stress among the groups in the thigh tissue. Similar to the thigh tissue, the MDA levels in the breast tissue do not exhibit statistically significant differences among the groups (*p* > 0.05). In the liver tissue, there are statistically significant differences among the groups (*p* < 0.05). Specifically, the control has the highest MDA level, while the HI-fed and ZM-fed groups have intermediate levels, and the TM-fed group has the lowest MDA level. This suggests that the TM-fed group has the lowest level of lipid peroxidation or oxidative stress in the liver, while the control exhibits the highest level. The differences between the HI-fed, ZM-fed, and control are less pronounced, with intermediate levels of MDA. In summary, results indicate that the TM-fed group has the lowest MDA levels in the liver, suggesting the least oxidative stress or lipid peroxidation in this tissue. However, there are no statistically significant differences in MDA levels among the groups in the thigh and breast tissues, indicating similar levels of oxidative stress or lipid peroxidation in these tissues across the experimental groups, including the ZM-fed group and the control.

In the thigh tissue, there are statistically significant differences in TPC among the groups (*p* < 0.001). The control, TM-fed, and HI-fed groups have similar TPC values. However, the ZM-fed group has a significantly lower TPC value compared to the other groups. This suggests that the type of insect used (ZM) had a lower TPC in the thigh tissue compared to the other groups. In the breast tissue, there are also statistically significant differences in TPC among the groups (*p* < 0.001). Groups control, TM-fed group, and ZM-fed group have similar TPC values. However, the HI-fed group has a significantly higher TPC value compared to the other groups, indicating that the type of insect used (HI) resulted in a significantly higher TPC in the breast tissue compared to the other groups. Finally, in the liver tissue, there are no statistically significant differences (*p* = 0.050). Groups control, TM-fed group, and ZM-fed group have similar TPC values, denoted by “b,” while the HI-fed group has a slightly higher TPC value, denoted by “a.” However, because the *p*-value is close to the significance threshold (0.05), it suggests that there might be some differences, but they are not strong enough to be considered statistically significant. In total, the type of insect used in the diet appears to have a significant impact on the TPC in the thigh and breast tissues, while the effect on liver tissue is not as clear-cut and does not reach statistical significance. These findings provide insights into how different insect species may influence the phenolic content in various tissues, which can have implications for the nutritional value and potential health benefits of insect-based diets.

In the thigh tissue, the control exhibited the highest TAC, while the TM-fed group, ZM-fed group, and HI-fed group had slightly lower TAC values. However, the differences in TAC among these groups were not statistically significant, as indicated by the *p*-value of 0.740. In the breast tissue, the control and HI-fed groups had relatively higher TAC values. The TM-fed group and ZM-fed group showed slightly lower TAC values. However, similar to the thigh tissue, the differences in TAC among these groups were not statistically significant (*p* = 0.907), indicating no significant variations in antioxidant capacity in the breast tissue. Finally, in the liver tissue, the control and ZM-fed groups exhibited lower TAC values. In contrast, the TM-fed group and HI-fed group had substantially higher TAC values. Although the liver tissue showed some variations in TAC among these groups, the *p*-value of 0.178 suggests that these differences were not statistically significant. Based on the phosphomolybdate method, there were no significant differences in TAC among the different groups in both thigh and breast tissues. However, in the liver tissue, there were some variations, but these differences were not statistically significant.

The DPPH levels in the thigh tissue among the four groups show little variation, with values ranging from approximately 15.11 to 17.42%. There are no significant differences in DPPH levels between these groups, as indicated by the *p*-value (0.946). Similar to the thigh tissue, the breast tissue also displays relatively consistent DPPH levels across the groups. DPPH levels range from around 13.72 to 17.04%, with no statistically significant differences detected (*p*-value of 0.791). In contrast to the thigh and breast tissues, the liver tissue exhibits higher DPPH levels across all groups, with values ranging from approximately 55.07 to 58.59%. However, similar to the other tissues, there are no statistically significant differences in DPPH levels among the groups (*p*-value of 0.942). Overall, the data indicate that the various insect-based diets (TM, HI, ZM) do not lead to significantly different levels of antioxidant activity in the examined tissues (thigh, breast, liver) compared to the control.

## Discussion

One significant stressor in animal husbandry is heat stress, stemming from an imbalance between the energy dissipated by the animal to its surroundings and the heat energy generated by the animal itself. This disbalance can arise from a mix of external circumstances that includes elevated temperatures, thermal radiation, air temperature, humidity, and airflow as well as animal-specific traits, such as species, metabolic rate, and thermoregulatory mechanisms. Environmental stressors like heat stress pose substantial challenges to animal agriculture (Nienaber and Hahn 2007; Renaudeau et al. 2012). Moreover, the public’s increased awareness and concern have placed environmental stress at the forefront of discussions in animal husbandry (Hyland et al. 2022).

The implications of heat stress on poultry production are profound, affecting both bird well-being and economic viability. High temperatures trigger a cascade of adverse effects in poultry, including reduced FI, diminished weight gain, and an increased risk of morbidity and mortality, as noted by Awad et al. (2020) and Barrett et al. (2019). Several studies have shown a negative impact on FI and BWG of broiler chickens, especially when the temperature exceeds 32 °C for prolonged periods of time (Goo et al. 2019; Imik et al. 2012; Khatlab et al. 2018). This trial showed higher BWG and FI for groups fed the treated diets at all growth stages, under a mild elevation of THI. Interestingly, FCR remained lower for each treated group compared to the control, indicating higher efficiency even under stress conditions. Differences between the starting and ending period of the thermal challenge did not affect mortality. Similar results were found by Kang et al. (2020) on laying hens, which were exposed to slightly elevated THI conditions. They also found that responses of broilers depend upon THI severity, and thus, a 7 °C increment of THI within 1 h lasting for over 4 h may impose a death rate of 100% on the birds.

Heat stress in broilers can also affect the oxidative status of the meat. It has been linked to increased oxidative stress in broiler chickens, which can have implications for the meat’s oxidative stability (Mancinelli et al. 2023). Oxidative stress is characterized by a reduction in the presence of antioxidants and an uneven equilibrium between the body’s ability to eliminate reactive oxygen species (ROS), which have the potential to harm muscle tissues. This oxidative imbalance can trigger lipid peroxidation, compromising meat quality in terms of color, flavor, and texture, ultimately affecting its shelf life due to increased susceptibility to spoilage and rancidity (Altan et al. 2003; Nawaz et al. 2021). Numerous trials have reported a negative impact of heat stress on poultry production; He et al. (2018) found that chronic heat stress alters the gene expression of appetite-related genes and hormones, negatively affecting both growth performance and intestinal morphology of broilers. Song et al. (2018) monitored a reduction in antioxidant genes and an almost doubled MDA value in the intestinal mucosa under heat stress, before improving it with the use of enzymatically treated *Artemisia annua.* Deterioration of the oxidative stability in broiler breast muscles was also monitored by El-Tarabany et al. (2021) when the birds were exposed in chronic heat stress.

The nutritional strategy followed is also of great importance; several different methodologies have been proposed in order to alleviate the destructive effects of heat stress. Studies have shown that chronic exposure to heat stress severely affects protein metabolism, which cannot be compensated through feed (Attia et al. 2018). Reducing protein consumption unavoidably leads to decreased feed efficiency. Proper amino acid intake, however, can potentially reduce the metabolic heat generated during digestion (Nawaz et al. 2021). Since insects like HI and TM have been explored for their balanced amino acid profile, which complements the dietary needs of broilers, they may offer a promising solution in alleviating heat stress in poultry.

Inclusion of insects in broiler diets can help mitigate the impact of heat stress on BW. Insects provide a highly digestible and balanced protein source, supporting muscle development even during periods of heat stress when traditional FI may be reduced (Verkerk et al. 2007). This comes in accordance with our findings, where insect-based feeds gave much heavier broiler chicken in comparison with the control, during the growing period. Notably, the HI-fed group consistently exhibited the highest BW throughout the experiment, signifying its influence on broiler growth under heat stress as a richer source of protein and fat compared to the larvae of the other insects. The mild heat stress conditions occurred during the last face of fattening possibly masked such a growth-promoting effect, if any. This finding suggests that HI larvae might contain nutritional components or bioactive compounds that enhance growth, potentially by mitigating the negative impacts of heat stress on nutrient utilization and metabolism.

Insects contain various bioactive substances that can have potential health benefits for animals (da Silva Lucas et al. 2020). These bioactive compounds can be found in different parts of insects, such as their exoskeleton, muscles, and other tissues. Among these compounds, chitin and chitosan stand out, derived from the insect exoskeleton, with applications in wound healing, drug delivery, and as potential cholesterol-lowering and prebiotic agents (Elieh Ali Komi et al. 2018). Insect proteins provide highly nutritious sources of essential amino acids, gaining attention as sustainable and alternative protein sources. In addition, insects contain lipids with essential fatty acids like omega-3 and omega-6, offering health benefits (Dobermann et al. 2017). Antioxidants, peptides, chitinase inhibitors, and phenolic compounds found in insects have been studied for their antimicrobial, antioxidant, and potential therapeutic properties (Nino et al. 2021). The results of this study indicate that insects have a positive impact on both BW and BWG, along with FI, especially during the early growth stages. This positive effect is likely due to the presence of various bioactive compounds that can enhance nutrient utilization and overall metabolic processes. Similar results have been found in several studies, with flavonoids and aromatic plant extracts promoting growth as well as reducing the oxidative stress in broilers (Yang et al. 2023, Zhang et al. 2021).

Furthermore, the bioactive compounds found in insects may also have implications for the oxidative status of broiler tissues, specifically the liver and muscle tissue. These compounds, including antioxidants and peptides, have been studied for their potential to mitigate oxidative stress (D’Antonio et al. 2021). Under heat stress conditions, broilers are particularly susceptible to oxidative stress, which can negatively impact their health and performance. Positive results have also been reported in studies with other feed additives containing antioxidant compounds; a study by Giannenas et al. (2022) indicated a positive impact through the addition of *Curcuma* and *Scutellaria* plant extracts on laying hen egg production as well as on egg oxidative stability. Farahat et al. (2016) found antioxidant and immunostimulant effects on broilers when feeding graded concentrations of green tea extract, without any negative effect on growth performance.

The presence of antioxidants in insect-based diets may help neutralize ROS and reduce oxidative damage in broiler tissues. Additionally, the peptides found in insects have been investigated for their antioxidant properties, which could contribute to maintaining the oxidative balance in vital organs like the liver and muscle tissue (Jakubczyk et al. 2020). As a result, broilers fed with insect-based diets may experience improved oxidative status, potentially leading to better overall health and performance.

The outcomes of this research provide valuable insights into the potential impact of supplementing broiler diets with insects on oxidative stress and antioxidant capacity, particularly when broilers face mild heat stress conditions. Our investigation into MDA levels, which serve as indicators of oxidative stress and lipid peroxidation, revealed intriguing findings. While the specific insect-based diet did not significantly influence MDA levels in thigh and breast tissues, a distinct effect was observed in the liver tissue. Notably, the group fed with TM larvae exhibited the lowest MDA levels in the liver, suggesting reduced oxidative stress and lipid peroxidation in this crucial organ. High MDA levels in the liver can impair metabolic functions, potentially leading to issues such as abnormal lipid metabolism (Ma et al. 2021). Tang et al. (2022) similarly found that chronic heat stress had detrimental effects on the growth, liver health, and redox status of broilers, with associated liver inflammation and oxidative stress. This underscores the potential of TM larvae as a dietary component in mitigating oxidative stress in the liver. The presence of bioactive compounds in insect-based diets appears to contribute to the improved oxidative status observed in broilers’ livers. These findings underscore the multifaceted benefits of insect-based diets in supporting broiler health and resilience under heat stress conditions.

An intriguing dimension in our study is the variation in TPC across thigh, breast, and liver tissues. The observed differences in TPC among the dietary groups suggest that the choice of insect species significantly influences the concentration of phenolic compounds in specific tissues. For example, the group fed with HI larvae demonstrated significantly higher TPC in the breast tissue. This variation could have implications for the nutritional value of broiler meat and potential health benefits associated with phenolic compounds. Our results suggest that the selection of insect species should be a carefully considered factor in diet formulation to achieve desired phenolic compound levels in broiler tissues. These findings align with those of Torres-Castillo and Olazarán-Santibáñez (2023), who discussed bioactive compounds, referred to as “entomochemicals,” in these insects, with a particular focus on phenolic compounds. Their study suggests that these edible insects can serve as a source of natural antioxidants due to the phenolic compounds they contain.

Furthermore, our data on TAC and DPPH levels did not reveal significant differences among the dietary groups in the examined tissues. This suggests that while the choice of insect-based diets influences TPC, it may not substantially impact antioxidant capacity. This could be attributed to a plethora of factors, including the presence of multiple antioxidant mechanisms that might buffer the impact of variations in TPC, making it challenging to observe significant differences in TAC. Additionally, phenolic compounds, while important, might not be the sole determinants of TAC. Their actions could be influenced by other compounds and antioxidants present in the tissues, leading to more nuanced and subtle effects that are not statistically significant (Flieger et al. 2021). Hence, the relationship between TPC and TAC is intricate, influenced by various factors, and may not exhibit straightforward linear correlations. While TPC is important for understanding the potential antioxidant content of a diet, it is just one piece of the puzzle in determining overall antioxidant capacity. Moreover, the complex nature of biological antioxidant systems can lead to non-significant differences when examining TAC and DPPH levels.

In conclusion, this experiment demonstrated a positive effect of insect meal inclusion in diets on broiler chickens when exposed to mild THI conditions. Growth performance was promoted, particularly when HI larvae were used, which are the richest in protein and fat content. Additionally, the presence of antioxidants in insect-based diets holds promise for enhancing broiler health and oxidative balance, especially when HI and TM are used. Overall, this study underscores the potential of insect-based diets in addressing the multifaceted challenges of oxidative stress and enhancing growth and the overall health and resilience of broilers. Nevertheless, further research is warranted to fully elucidate the underlying mechanisms and practical implications of these findings.

## Data Availability

Data described in the manuscript will be made publicly and freely available without restriction.
